# The objective structured clinical examination in family medicine training: A comprehensive review for the South African context

**DOI:** 10.4102/safp.v68i2.6256

**Published:** 2026-02-23

**Authors:** Selvandran Rangiah

**Affiliations:** 1 Department of Family Medicine, College of Health Sciences, University of KwaZulu-Natal, Durban, South Africa

**Keywords:** OSCE, review, history, challenges, medical education

## Abstract

The valid and reliable assessment of clinical competence is a cornerstone of medical education. The objective structured clinical examination (OSCE) has been the hallmark of performance-based assessments for decades. A narrative review of foundational and contemporary literature, encompassing educational theory, psychometric studies and policy documents, was conducted to explore the OSCE’s historical, theoretical and practical dimensions, focusing on its application, challenges and evolution within South African family medicine training. The article synthesises the OSCE benefits, its role in the fellowship examination and discusses the evolution towards a programmatic assessment incorporating workplace-based assessment. The OSCE remains a vital tool in medical education, but its role is evolving. Its future relevance will depend on continuous refinement, technological integration and thoughtful positioning alongside other assessment modalities.

**Contribution:**

This article provides a consolidated, context-specific resource for family physicians, registrars and educators, critically appraising the OSCE’s enduring value and future trajectory within the local training landscape.

## Introduction

### The persuit of objective clinical assessment

The fundamental mandate of medical education is to ensure graduates possess the requisite clinical competence to provide safe and effective healthcare.^1^ This is particularly critical in family medicine, where practitioners must manage a wide spectrum of undifferentiated problems across all age groups. Consequently, the methods used to assess this competence must be robust, fair and defensible. For much of the 20th century, clinical assessment relied on traditional methods such as the unstructured oral examination, or ‘viva’, and the ‘long case’, where a candidate would be assessed on a single patient.^2^ While these methods allowed for assessing depth and clinical reasoning, a growing body of evidence highlighted significant issues with their validity, fairness and psychometric reliability.^3,4,5^ A candidate’s performance with a single patient was found to be a poor predictor of performance with another, and the lack of standardisation meant that candidates were often not assessed on comparable challenges, leading to inconsistency and bias in grading.^3,6^

This growing dissatisfaction with the subjectivity and psychometric weaknesses of traditional methods prompted a paradigm shift in clinical assessment. In the 1970s, Harden and colleagues introduced the objective structured clinical examination (OSCE) as a landmark innovation aimed at addressing these shortcomings.^7,8^ The OSCE was defined as ‘an approach to the assessment of clinical competence in which the components were assessed in a planned or structured way with attention being paid to the objectivity of the examination’.^9^

This review aims to provide a comprehensive and critical analysis of the OSCE, from its theoretical underpinnings to its practical implementation. It will focus on the dual value of the OSCE: as a summative assessment of learning (for certification) and a formative assessment for learning (for feedback and development). Critically, this analysis will situate itself within the unique context of postgraduate family medicine training in South Africa, examining the OSCE’s integral role in university programmes and the national Fellowship of the College of Family Physicians of South Africa (FCFP-SA) examination.

## Historical development and theoretical foundations

### A response to a need

The development of the OSCE in the mid-1970s was a direct response to the increasing application of measurement theory to educational assessment.^2,9^ Traditional clinical examinations, heavily reliant on subjective expert judgement, were found wanting when scrutinised through psychometrics. The seminal papers by Harden et al.^7^ and Harden and Gleeson^8^ formally introduced a new methodology built on the principles of standardisation and objectivity. The OSCE format rapidly gained global acceptance, where candidates rotate through a circuit of stations to perform specific tasks on standardised patients (SPs) or simulators.^10^ It is now widely used across undergraduate, postgraduate and licensure contexts in virtually every medical discipline.^10,11^

### Educational theory: The ‘why’ behind the ‘how’

The OSCE’s enduring success stems from its structure and its grounding in a robust framework of interconnected educational theories. This section explores the key theories underpinning the OSCE: constructivism, social constructivism, authentic assessment, experiential learning and competency-based education (CBE).

At its core, the OSCE aligns with constructivism, the theory which posits that learners actively construct their knowledge through experience rather than passively receiving it.^12,13^ An OSCE station is more than a test; it is a learning event where candidates must recognise, retrieve, apply and integrate their pre-existing knowledge and experience to solve a clinical problem. This active process of knowledge construction is often facilitated through interaction with SPs, peers and assessors, invoking the principles of social constructivism, where meaning is co-created through collaborative learning, dialogue and feedback.^14^ This is essential for learners’ professional development.

This process is designed to be an authentic assessment, which evaluates a learner’s ability to perform tasks that closely resemble professional practice.^15^ By simulating clinical encounters, the OSCE assesses competencies in a controlled, yet realistic environment. This directly links to experiential learning, where performing a task (at the station), receiving structured feedback and engaging in self-reflection drive deeper understanding and skill development.^12^

Furthermore, the OSCE is a natural fit for CBE, which focuses on demonstrating specific, predefined abilities required within effective practice.^16,17^ The station-based design allows for the targeted assessment of discrete competencies – such as communication, procedural skills or clinical reasoning – that form the building blocks of a competent family physician. Performance is measured using criterion-referenced assessment, meaning candidates are evaluated against a predetermined performance standard (e.g. a checklist or scoring rubric) rather than being ranked against their peers.^6,15^ This principle is fundamental to the OSCE’s objectivity and fairness.

### Philosophical underpinning: Pragmatism

While grounded in educational theory, the OSCE is also influenced by the philosophical perspective of pragmatism. Pragmatism is a tradition that links theory and practice, emphasising the practical consequences and usefulness of ideas and actions.^18,19^ It values what ‘works’ in solving real-world problems. The OSCE is inherently pragmatic in its design and purpose. It moves beyond assessing what a candidate knows to what they can do, focusing on the practical application of theoretical knowledge in realistic scenarios.^20^ The value of the OSCE is judged by its utility in preparing competent healthcare professionals for the complexities of clinical practice. This focus on problem-solving, contextualised learning and observable actions aligns perfectly with pragmatism’s emphasis on the practical effectiveness of education and assessment.^21,22^

## Core principles and design of a modern objective structured clinical examination

### The blueprint: Structure and components

A well-designed OSCE is a logistically complex undertaking built upon several core components that work together to ensure a standardised and effective assessment. These components are discussed as follows:

- **Stations:** The examination consists of a circuit of timed stations, each typically lasting 5–15 min.^6,23^ A key principle for ensuring reliability is the use of multiple stations to sample a wide range of clinical skills and content areas.^6^
- **Station types:** Stations are designed to assess different competencies and can include history-taking, physical examination, procedural skills (e.g. suturing, joint injection), communication challenges (e.g. breaking bad news, counselling) and data interpretation (e.g. reviewing X-rays or laboratory results).^24^
- **Scenarios and standardised patients:** Central to the OSCE are realistic clinical scenarios and meticulously trained SPs (also known as simulated patients). Standardised patients are trained to portray a specific case consistently for all candidates, including the medical history, physical findings and emotional responses.^25^ This standardisation is critical for fairness and realism.^26^
- **Assessors and training:** Trained assessors, often academics or clinical experts, observe and score candidate performance at each station. To minimise inter-rater variability – a significant threat to reliability – assessors must be thoroughly trained on using the scoring instruments and calibrated to ensure consistent judgement.^27^ The use of multiple different assessors across the circuit helps to mitigate the impact of any single examiner’s potential bias.^2^ Employing multiple examiners for a single station can enhance reliability and reduce individual bias.
- **Scoring rubrics:** Performance is evaluated using predetermined scoring criteria, such as objective checklists or global rating scales. Checklists itemise the specific actions a candidate is expected to perform, while global rating scales allow the examiner to judge performance more holistically against a descriptive scale.^24^ These rubrics must be clear, transparent and directly linked to the learning objectives of the station.

### The psychometric cornerstones: Validity and reliability

The credibility of any high-stakes examination rests on its psychometric properties, primarily its validity and reliability.

Validity refers to the degree to which an examination measures what it purports to measure. Objective structured clinical examinations strive for high content validity by carefully blueprinting the stations against the curriculum and the expected competencies of a practitioner in the field.^6,28^ This ensures the content is relevant and representative. Construct validity – the extent to which the OSCE truly measures the complex construct of ‘clinical competence’ – is more debated. Critics argue that performance in a simulated setting may not perfectly translate to performance in real-world clinical practice.^2,23^

Reliability refers to the consistency and reproducibility of the assessment scores.^29^ If a candidate were to take an equivalent test on another day, they should receive a similar score. The reliability of an OSCE is enhanced by three key factors: having a sufficient number of stations (to ensure adequate sampling of skills), using multiple trained examiners and ensuring standardisation of scenarios and SPs.^6,28^ Advanced statistical methods, such as Generalisability Theory, can be used to analyse the various sources of measurement error (e.g. variation because of candidates, stations or examiners) and to optimise the examination’s design for reliability.^29^

In designing an OSCE, a fundamental tension exists between maximising standardisation for reliability and enhancing authenticity for the sake of validity. A highly standardised station using a mannequin for procedural skills may yield consistent scores, but it lacks the fidelity of a real patient encounter. Conversely, a highly authentic and complex station with an SP may introduce more performance variability, slightly reducing reliability. The art of effective OSCE design lies in finding a pragmatic balance between these competing demands, tailoring the approach to the specific purpose and stakes of the examination.

## A critical appraisal: Benefits and challenges

Despite its widespread adoption, the OSCE is not without its critics. A balanced evaluation reveals both significant strengths and considerable challenges, which are summarised in Table 1.

**TABLE 1:** Summary of the benefits and challenges of the objective structured clinical examination.

Benefits	Challenges
High reliability and objectivity	Resource-intensive (cost, personnel, time)
Comprehensive sampling of clinical skills	Limited authenticity and construct validity
Provides structured feedback for learning	Induces high levels of candidate anxiety and stress
Standardised format ensures fairness	Potential for examiner and SP variability
Serves as a robust quality assurance tool	Promotes fragmentation of holistic clinical tasks
Encourages active, self-directed learning	Logistical complexity in organisation

Note: Information based on this article’s references.^1,2,7^

SP, standardised patient.

### Established benefits: The dual value

The primary advantage of the OSCE lies in its objectivity and reliability. The structured format, standardised tasks and criterion-referenced scoring rubrics are designed to minimise examiner subjectivity and bias, leading to fairer and more consistent results than traditional methods.^2,26,30^ The multi-station format allows for a comprehensive assessment by sampling a broad range of clinical skills, providing a more holistic view of a candidate’s competence than a single patient encounter can offer.^23^

Objective structured clinical examinations serve a crucial quality assurance function for educational programmes, ensuring that graduates meet a minimum standard of competence before they are certified for independent practice.^2^

However, its value extends beyond its summative role of ensuring candidates meet the necessary standards to pass an examination. The OSCE serves a critical formative function in health professions education as a powerful tool for feedback and learning. They provide candidates with specific, targeted feedback on their performance, highlighting areas for improvement early in their training. This feedback-driven approach promotes self-directed learning and reflective practice, especially when OSCEs are used for formative purposes.^31,32^ Through this feedback-driven improvement cycle, the OSCE functions not just as an assessment of learning, but also as an assessment for learning, helping students learn from mistakes in real time and guiding them towards better clinical practice.

Low-stakes formative OSCEs help reduce anxiety and build confidence as learners have the opportunity to improve their performance through guided practice and remediation before high-stakes summative assessments.

### Significant challenges

The most frequently cited drawback of the OSCE is that it is highly resource-intensive. Implementing a high-quality OSCE is expensive and logistically demanding, requiring substantial investment in physical space, equipment, administrative support and personnel for training SPs and examiners.^2,11,33^ Another primary concern relates to authenticity and validity. The artificial, time-constrained environment of an OSCE station may not fully capture the complexity, uncertainty and longitudinal nature of real clinical practice.^23,24^ The validity heavily relies on the writing of good, real-life scenarios for the stations. This raises valid questions about how well performance in an OSCE predicts performance in the actual workplace.

The high-stakes nature of the summative examination can induce significant candidate anxiety and stress, which may negatively affect performance and not be a true reflection of the candidate’s underlying ability.^27,31,34^ Despite efforts to standardise, examiner and SP variability can remain challenging. Differences in examiner stringency or interpretation of rubrics can introduce inconsistencies, and recruiting and training a diverse pool of SPs to represent the full spectrum of patient populations can be difficult.^27,35^ Lastly, by deconstructing clinical encounters into discrete tasks, the OSCE can be criticised for promoting a fragmentation of care. This approach may assess skills in isolation and fail to capture the holistic, integrated nature of a family physician’s work, where multiple issues are often managed concurrently.^23,36^

### The objective structured clinical examination in South African family medicine: From university to fellowship

In South Africa, the OSCE is a cornerstone of postgraduate family medicine training, serving both formative and summative purposes. It is utilised by all nine university departments that train specialist family physicians and forms the definitive clinical component of the national Fellowship examination.^6^

### The high-stakes gateway: Fellowship of the College of Family Physicians of South Africa Examination

The summative assessment of a family physician’s clinical skills for specialist registration is the FCFP-SA examination, administered by the Colleges of Medicine of South Africa (CMSA). The clinical component of this high-stakes examination consists entirely of OSCEs.^37^ Candidates are invited to the OSCE only after successfully passing a series of written papers covering multiple-choice questions, short-answer questions and a critical appraisal of research.^37,38^ The OSCEs are designed to assess a candidate’s clinical and consultation skills against the standard expected of a competent specialist family physician.^37^ The content is blueprinted against the five national unit standards for Family Medicine training, which cover clinical governance, clinical practice, community-oriented care, teaching, and professionalism.^38,39^

### University-level implementation and standard-setting innovation

At the university level, OSCEs are used within undergraduate and postgraduate programmes for formative assessment (to drive learning and provide feedback) and summative assessments (for progression). South Africa has made a significant contribution to the field of OSCE methodology, particularly in standard setting.^6^

Recognising that a fixed 50% pass mark is an arbitrary figure that may not accurately represent the required level of competency, local educators have pioneered more defensible methods.^6^ Work led by Mash at Stellenbosch University introduced and validated the borderline regression method for the family medicine OSCE.^6^ This method requires examiners to make two separate judgements for each candidate at each station: firstly, a detailed score using the objective checklist, and secondly, a holistic global rating of the candidate’s overall performance (e.g. ‘clearly a family physician’, ‘borderline/not sure’, or ‘clearly not a family physician’). After the examination, the checklist scores for all candidates are statistically regressed against their global ratings to determine the pass mark.^6^ While this data-driven approach is considered highly credible, a key limitation is its reliance on the high number of candidates to ensure statistical reliability.^40,41^

Because of the smaller candidate cohorts often found in South African postgraduate examinations, the modified Angoff method is currently used for standard setting.

The Angoff method is a well-established, test-centred approach where a panel of experts evaluates each assessment item to estimate the score a ‘minimally competent’ candidate would likely achieve.^42,43^ This method is considered justifiable and feasible for high-stakes examinations, particularly when modifications such as group discussion and review of candidate performance data (a ‘reality check’) are included to improve reliability and defensibility. This pragmatic choice ensures that a robust, criterion-referenced standard is maintained even with limited numbers of candidates.^44^

## The evolving landscape and future directions

The role of the OSCE in clinical assessment is not static. It is evolving in response to technological advancements and a deeper understanding of the principles of effective assessment.

### The technological shift

Technology is increasingly being integrated into the OSCE. The most significant recent development has been the widespread adoption of virtual OSCEs (vOSCEs), largely accelerated by the coronavirus disease 2019 (COVID-19) pandemic that served as a catalyst for rapid innovation.^41^ Using video conferencing platforms, candidates can interact with SPs remotely, offering greater flexibility, a wider geographic reach, and enhanced safety during public health crises.^2,41^ To ensure the continuation of high-stakes examinations, the College of Family Physicians successfully transitioned to conducting online or vOSCEs, demonstrating the adaptability of the assessment system in response to unprecedented challenges.^45^

However, vOSCEs have significant limitations, most notably the inability to directly assess physical examination and procedural skills, which restricts the range of competencies that can be evaluated.^35^

Other new technological enhancements are also emerging, such as high-fidelity mannequins that simulate realistic physiological responses and digital scoring platforms that streamline data collection and provide faster feedback to candidates.^2^

### Beyond the objective structured clinical examination: Programmatic assessment in South Africa

A significant contemporary movement in medical education is the shift towards programmatic assessment. This model moves away from reliance on single, high-stakes assessment events and instead bases judgements of competence on the synthesis of multiple data points collected over time in various settings.^46^ In line with this global trend, the CMSA has mandated the integration of workplace-based assessment (WPBA) into all specialist training programmes in South Africa.^46,47^

This shift is a direct and pragmatic response to the acknowledged limitations of the OSCE, particularly its lack of authenticity. While the OSCE assesses competence at the ‘shows how’ level of Miller’s Pyramid (demonstrating a skill in a controlled, simulated setting), WPBA assesses performance at the ‘does’ level – in the real, unpredictable workplace context.^46,47^ To facilitate this, South African family medicine has developed a national set of Entrustable Professional Activities (EPAs). Entrustable Professional Activities are units of professional practice (e.g. ‘manage a district hospital theatre list’) that can be entrusted to a trainee for independent execution once sufficient competence has been demonstrated through repeated observation in the workplace.^46^

This evolution does not render the OSCE obsolete. Instead, it repositions it as one crucial component within a more comprehensive assessment system. The OSCE’s strength lies in its ability to provide reliable, standardised data on a broad range of foundational clinical skills. This data can complement the rich, authentic and longitudinal evidence of integrated performance gathered through WPBA and EPAs. The future of assessment in South African family medicine lies in this blended model, which balances the reliability of the OSCE with the authenticity of workplace performance to create a more holistic and valid system.

## Conclusion

The OSCE emerged as a revolutionary tool that brought much-needed objectivity, standardisation and psychometric rigour to clinical assessment. Its benefits in providing a fair and comprehensive evaluation of a wide range of clinical skills have cemented its place as a global standard in medical education. However, it is not a panacea. The OSCE faces significant challenges related to resource intensity, candidate stress and a persistent tension between standardisation and the authenticity of real-world clinical practice.

In South Africa, the OSCE is an integral component of family medicine training (used for both formative training and summative certification), from undergraduate and postgraduate programmes to the national FCFP-SA fellowship examination. Local educators have adopted the OSCE and contributed to its refinement through sophisticated standard-setting methods that enhance the credibility of its outcomes. The future of the OSCE, both locally and internationally, lies not in its isolated use but in its thoughtful integration into a programmatic assessment system. Its enduring value will be as a provider of reliable, standardised data on core competencies, which, when triangulated with evidence from authentic workplace-based assessments such as EPAs, creates a more robust, defensible and holistic system for training and certifying the competent, practice-ready family physicians that South Africa’s health system requires.
